# Contagious: Cultures, Carriers, and the Outbreak Narrative

**DOI:** 10.3201/eid1502.081408

**Published:** 2009-02

**Authors:** Stephen B. Thacker

**Affiliations:** Centers for Disease Control and Prevention, Atlanta, Georgia, USA

**Keywords:** disease emergence, outbreak narrative, social relationships, book review

The outbreak narrative, which tells the evolving story of disease emergence, is the central theme of this book by Priscilla Wald, an English professor at Duke University. Wald discusses and challenges outbreak narratives that use a formulaic plot line: identification of an emerging infection, discussion of global networks through which diseases travel, and a chronicle of the epidemiologic work that results in disease containment. The expectation created by that formula, Wald contends, has at times hampered our ability to address such emerging diseases as HIV infection because we distort or exaggerate the story to fit the formula. Possibly more important, she claims, is the development of the outbreak narrative in the late 19th century, which coincided with emergence of urban sociology and the concept of social contagion. During this time, circulation of ideas and attitudes turned individuals into social groups and cultures, and terms such as contagion and infection entered lay language.

Wald describes Mary Mallon, “Typhoid Mary,” as the prototypical healthy carrier whose outbreak narrative led to a new way of thinking about social relationships and social responsibility. Her status as an immigrant contributed to society’s blaming and stigmatizing immigrants because of their association with communicable disease (especially venereal disease), a stigma reanimated in the 1980s with the emergence of HIV and its most infamous carrier, Patient Zero.

Wald’s analysis of disease emergence also notes its simultaneous evolution with social, religious, and political changes. The viral metaphor was applied to biologic warfare in the 1950s and expanded to describe political contagion. According to J. Edgar Hoover, “the bloody virus of communism” was spread by agents (carriers) trying to “infiltrate and colonize this country.”

The microbe is the worthy foe in epidemiologists’ stories, which can be seen as analogous to good detective stories that have happy endings and draw attention to urgent problems. However, Wald warns, what makes the story appealing can distort articulation of the problem. The danger, she believes, lies in the storytelling, not in the epidemiology or laboratory science. Wald also notes that epidemiology’s shift away from infectious diseases beginning in the 1960s removed the heroic edge from the field. She writes, “Sociology did not make for risk, exciting disease detectives.” Wald is wary of the limitations of the outbreak narrative and advocates a model for global health based on social justice.

The book is academic in presentation, and Wald’s conclusions are not always compelling. For example, her discussion of epidemiologic horror in books and movies such as Invasion of the Body Snatchers will intrigue some readers and put off others who might find this analysis extreme and detracting from the serious tone of the rest of the book. Still, the thesis involving the outbreak narrative is interesting, and readers intrigued by the influence of epidemiology on emerging infections as well as its broader implications to society and the world will find this book worthwhile.

